# Differential effect of SARS-CoV-2 infection on stress granule formation in Vero and Calu-3 cells

**DOI:** 10.3389/fmicb.2022.997539

**Published:** 2022-08-23

**Authors:** Dongbum Kim, Sony Maharjan, Mijeong Kang, Jinsoo Kim, Sangkyu Park, Minyoung Kim, Kyeongbin Baek, Suyeon Kim, Jun Gyo Suh, Younghee Lee, Hyung-Joo Kwon

**Affiliations:** ^1^Institute of Medical Science, College of Medicine, Hallym University, Chuncheon, South Korea; ^2^Department of Microbiology, College of Medicine, Hallym University, Chuncheon, South Korea; ^3^Department of Biochemistry, College of Natural Sciences, Chungbuk National University, Cheongju, South Korea; ^4^Department of Medical Genetics, College of Medicine, Hallym University, Chuncheon, South Korea

**Keywords:** SARS-CoV-2, COVID-19, sodium arsenite, G3BP1/2, N protein, stress granule

## Abstract

Stress granule formation is induced by numerous environmental stressors, including sodium arsenite treatment and viral infection. Accordingly, stress granules can inhibit viral propagation and function as part of the antiviral host response to numerous viral infections. Severe acute respiratory syndrome coronavirus-2 (SARS-CoV-2) antagonizes stress granule formation, in part, via interaction between SARS-CoV-2 nucleocapsid (N) protein and Ras-GTPase-activating SH3-domain-binding protein 1 (G3BP1). However, it is unclear whether there are differential effects in different cell types. In this study, we assessed interaction between the N protein of SARS-CoV-2 S clade and G3BP1/2 in Vero and Calu-3 cells and investigated the effect of various SARS-CoV-2 strains on sodium arsenite-induced stress granule formation. Our data show that SARS-CoV-2 S clade N protein interacts with both G3BP1 and G3BP2 more strongly in Calu-3 *vs*. Vero cells. Consistent with this observation, infection with SARS-CoV-2 S clade induces stress granule formation in Vero but not in Calu-3 cells. However, infection with SARS-CoV-2 S clade, as well as other SARS-CoV-2 variants, inhibits sodium arsenite-induced stress granule formation in both cell lines. Taken together, our results show differential effects of SARS-CoV-2 infection on stress granule formation that is dependent on host cell type, rather than virus strain type.

## Introduction

The devastating coronavirus disease 2019 (COVID-19) pandemic, which is caused by severe acute respiratory syndrome coronavirus-2 (SARS-CoV-2), continues to spread, posing a tremendous threat to both public health and economic stability worldwide (Chen et al., 2020; Huang et al., 2020; Zhou et al., 2020). SARS-CoV-2, like other coronaviruses, is an enveloped, positive-sense single-stranded RNA virus with a genome of ∼30,000 nucleotides (Lu et al., 2020) encoding four structural proteins: spike (S), membrane (M), envelope (E), and nucleocapsid (N) (Wu et al., 2020; V’kovski et al., 2021). Beyond vaccine development, most studies on SARS-CoV-2 to date have focused on identifying strategies to reduce the pathophysiology of infection by suppressing viral replication inside the host (Blake et al., 2021; Zhang et al., 2021). However, while these investigations have led to the development of antiviral therapies for COVID-19 (*i.e.*, ritonavir-boosted nirmatrelvir, sold under the commercial name Paxlovid), mutations that reduce drug efficacy have been identified in *in vitro* selected SARS-CoV-2 variants, highlighting the need for new antiviral strategies to emerging SARS-CoV-2 variants (Szemiel et al., 2021; Stevens et al., 2022).

In response to viral infection, cells can initiate several molecular and physiological processes aimed at combatting the infection and suppressing viral replication (Sumbria et al., 2021). These include the induction of stress responses and the formation of stress granules within infected cells. Stress granules are dense membrane-less ribonucleoprotein (RNP) complexes that dynamically assemble in the cytoplasm of cells exposed to environmental stressors, such as ultraviolet radiation, heat and cold shock, nutrient starvation, sodium arsenite treatment, and viral infection (White and Lloyd, 2012; Protter and Parker, 2016; Mahboubi and Stochaj, 2017). They form from the accumulation of translationally inactive mRNAs and stalled translation initiation complexes, and their primary function is to promote cell survival (Ohn et al., 2008; Ivanov et al., 2019; Campos-Melo et al., 2021).

In particular, previous studies have shown that stress granules can suppress viral replication and function as a key part of the antiviral host response to various viral pathogens (Onomoto et al., 2012; McCormick and Khaperskyy, 2017). Stress granule formation in virus-infected cells is initiated when double-stranded (ds)RNA or single-stranded (ss)RNA, common viral replication intermediates, bind to protein kinase R (PKR), triggering its autophosphorylation and activation (Cesaro and Michiels, 2021). Once activated, PKR phosphorylates the eukaryotic translation initiation factor (eIF2α) and triggers assembly of stress granules by the nucleating proteins Ras-GTPase-activating SH3-domain-binding protein 1 (G3BP1) and T-cell-restricted intracellular antigen-1 (TIA-1) (Gilks et al., 2004; Matsuki et al., 2013). Phosphorylated eIF2α further prevents formation of tRNA^Met^-GTP–eIF2 complexes, which inhibits viral mRNA translation, ultimately blocking viral replication (Liu et al., 2020).

In response to this stress granule-mediated inhibition, viruses have evolved various mechanisms to antagonize stress granule formation and thereby enhance viral replication (Rabouw et al., 2016; Hou et al., 2017; Nakagawa et al., 2018). For example, hepatitis C virus and Japanese encephalitis virus inhibit antiviral stress granule formation by targeting PKR (Toroney et al., 2010; Tu et al., 2012), whereas others, including Zika virus, Junin virus, and West Nile virus, block assembly of stress granules by modulating the eIF2α-dependent pathway (Linero et al., 2011; Amorim et al., 2017; Basu et al., 2017). Alternatively, enterovirus and foot-and-mouth disease virus produce a viral protease known as 3C^pro^, which cleaves G3BP1 at amino acids Q326 and E284, leading to disruption of stress granule formation and enhanced viral replication (Ye et al., 2018; Zhang et al., 2018). Likewise, strategies to modulate or inhibit stress granule formation have also been reported for diverse groups of coronaviruses. In Middle East respiratory syndrome coronavirus (MERS-CoV), the virus-encoded protein 4a sequesters viral RNA and blocks binding to PKR, preventing its downstream activation (Rabouw et al., 2016; Nakagawa et al., 2018). In contrast, another coronavirus—infectious bronchitis virus—can inhibit stress granule formation by modulating both eIF2α-dependent and eIF2α-independent signaling pathways (Brownsword et al., 2020).

Notably, a recent study reported that similar to MERS-CoV 4a protein, the SARS-CoV-2 N protein binds to PKR and prevents its autophosphorylation (Zheng et al., 2021). However, in addition, N protein was also found to interact with and inhibit G3BP1, thus impairing stress granule formation via two distinct mechanisms (Zheng et al., 2021). These observations are supported by another study, which found that SARS-CoV-2 N protein inhibits host stress granule by interacting with G3BP1 and G3BP2 and sequestering these proteins from their interacting partners (Nabeel-Shah et al., 2022). However, given the wide cell tropism exhibited by SARS-CoV-2 and the rapidly changing genetic landscape of this virus, it is unclear how the extent of stress granule inhibition varies in different cell types and or with distinct viral variants. Here, to address these questions, we utilized our previously developed anti-N protein antibody (Kim et al., 2021a) to measure the binding between cellular G3BP1 and N protein from SARS-CoV-2 S clade, as well as the subsequent inhibition of stress granule formation, in Vero and Calu-3 cells. In addition, we determined how infection with different SARS-CoV-2 variants affects stress granule formation in response to viral infection and sodium arsenite treatment. Through this study, we aim to better understand the mechanisms by which SARS-CoV-2 blocks antiviral responses to enhance viral replication and promote pathogenesis.

## Materials and methods

### Cell and virus culture

The African green monkey kidney Vero and Vero E6 cell lines, as well as the human airway epithelial Calu-3 cell line, were obtained from the Korean Cell Line Bank (Seoul, South Korea). Cells were grown in Dulbecco’s Modified Eagle Medium (DMEM, Thermo Fisher Scientific, Waltham, MA, United States), containing 10% fetal bovine serum (FBS; Thermo Fisher Scientific), 25 mM HEPES, 100 U/mL penicillin, and 100 μg/mL streptomycin, and maintained at 37°C in a 5% CO_2_ incubator.

SARS-CoV-2 S clade (hCoV-19/Korea/KCDC03/2020, lineage A, NCCP43326), alpha variant (hCoV-19/Korea/KDCA51463/2021, lineage B.1.1.7, 501Y.V1, NCCP43381), delta variant (hCoV-19/Korea/KDCA119861/2021, lineage B.1.617.2, NCCP43390), mu variant (hCoV-19/Korea/KDCA159392/2021, lineage B.1.621, NCCP43407), and omicron variant (hCoV-19/Korea/KDCA447321/2021, lineage B.1.1.529, NCCP43408) were provided by the National Culture Collection for Pathogens (Osong, South Korea).

SARS-CoV-2 viruses were amplified in Vero cells; virus titers in cell culture supernatants were quantified by plaque assays, as described below, and viral stocks (1 × 10^7^ pfu/mL) were stored at -70°C. SARS-CoV-2 amplification and cell culture infection experiments were performed under biosafety level 3 (BSL-3) conditions in the Research Institute of Medical-Bio Convergence of Hallym University. BSL-3 protocols were approved by the Institutional Biosafety Committee of Hallym University (Permit no. Hallym2020-12, 2022-03).

### Virus titration by plaque assay

To estimate viral titers, we performed plaque assays on Vero E6 cells, as described previously (Kim et al., 2021b,2022a; Park et al., 2021a). In brief, Vero E6 cells (7 × 10^5^ cells/well) were cultured in 6-well plates (Corning Inc., Corning, NY, United States) for 12 h. Cells were then washed with phosphate-buffered saline (PBS) and infected with 10-fold serial dilutions of virus culture supernatants. After 1 h adsorption with shaking every 10 min, supernatants were removed, and the wells were covered with 3 mL DMEM/F12 medium (Thermo Fisher Scientific), containing 2% Oxoid agar and N-*p*-Tosyl-L-phenylalanine chloromethyl ketone (TPCK; 1 μg/mL)-treated trypsin. Plates were incubated at 37°C for 3 days and stained with 0.1% crystal violet for 1 h to visualize plaque formation. Viral titers were then determined by counting the number of plaques.

### Antibodies

Monoclonal antibody against the SARS-CoV-2 N protein (anti-SARS-CoV-2 N mAb) was prepared from ascitic fluid collected from BALB/c mice injected with hybridoma cells (clone 1G10C4 mAb), as previously described (Kim et al., 2021a,2022a,2022b). Rabbit anti-SARS-CoV-2 N protein polyclonal antibody (anti-SARS-CoV-2 N Ab, Catalog no. 40588-T62) was purchased from Sino Biological (Beijing, China), rabbit anti-G3BP1 antibody (Catalog no. 61559S) was purchased from Cell Signaling (Danvers, MA, United States), rabbit anti-G3BP2 antibody (Catalog no. MB S852913) was purchased from MyBioSource (San Diego, CA, United States), and anti-β-actin antibody was obtained from Sigma-Aldrich (St. Louis, MO, United States). Alexa Fluor 546-conjugated goat anti-mouse IgG (h + L) antibody (Catalog no. A11030) and Alexa Fluor 488-conjugated goat anti-rabbit IgG (h + L) antibody (Catalog no. A11008) were purchased from Thermo Fisher Scientific.

### SARS-CoV-2 infection and co-immunoprecipitation experiments

Calu-3 cells were cultured for 18 h at a density of 3 × 10^5^ cells/well in 6-well plates. Cells were washed in PBS and infected with SARS-CoV-2 (S clade) at 0.1 multiplicity of infection (MOI) for 1 h, with shaking every 15 min, to allow adsorption of the virus, in a 5% CO_2_ incubator at 37°C. The supernatants were then aspirated and replaced with DMEM, containing 2% FBS. After 72 h infection, cells were washed with PBS and lysed for 30 min at 4°C in cell-lysis buffer (10 mM HEPES, 150 mM NaCl, 5 mM EDTA, 100 mM NaF, 2 mM Na_3_VO_4_, protease inhibitor cocktail, and 1% NP-40). Cell lysates were collected by centrifugation at 14,000 rpm at 4°C for 20 min and then incubated with anti-SARS-CoV-2 N mAb and Protein A beads (CaptivA^tm^ PriMAB 52% [v/v] slurry; REPLIGEN, Waltham, MA, United States) for 2 h at 4°C. Anti-SARS-CoV-2 N mAb and Protein A bead immunocomplexes were collected by centrifugation and resolved by 4–12% gradient sodium dodecyl sulfate-polyacrylamide gel electrophoresis (SDS-PAGE) using Bolt™ 4–12% Bis–Tris Plus gels (Thermo Fisher Scientific). Co-IP gels were stained with Coomassie brilliant blue G-250 or analyzed by western blot, as described below.

### Mass spectrometry to identify SARS-CoV-2 N protein-binding partners

After Co-IP with anti-SARS-CoV-2 N mAb, the protein bands of interest identified by 4–12% gradient SDS-PAGE were excised from gels and analyzed by Proteinworks Co (Seoul, South Korea), as described previously (Park et al., 2021b). In brief, protein was digested with trypsin, and the resulting peptides were isolated using an Acclaim™ PepMap™ 100 C18 LC Column (Thermo Fisher Scientific). These peptides were examined by tandem mass spectrometry (MS/MS), using a Q Exactive™ Plus Hybrid Quadrupole-Orbitrap™ Mass Spectrometer (Thermo Fisher Scientific). Peptide sequences were then analyzed, and corresponding proteins were identified using the National Center for Biotechnology Information (NCBI)^1^ database. The mass spectrometry proteomics data have been deposited to the ProteomeXchange Consortium via the PRIDE (Perez-Riverol et al., 2022) partner repository with the dataset identifier PXD035715.

### Western blot analysis

Vero and Calu-3 cells infected with SARS-CoV-2 and uninfected control cells were lysed with cell-lysis buffer, and cell lysates were collected by centrifugation at 14,000 rpm at 4°C for 20 min. Proteins were resolved on 4–12% Bis–Tris gradient gels and transferred onto nitrocellulose membranes. These were blocked in PBS with 0.1% Tween-20 (PBST), containing 5% w/v non-fat dry milk, and then incubated with anti-SARS-CoV-2 N mAb, anti-G3BP1 antibody, anti-G3BP2 antibody, or anti-β-actin antibody overnight at 4°C. Membranes were washed in PBST and incubated with a horseradish peroxidase-conjugated secondary antibody, and the immunoreactive bands were developed and visualized using an enhanced chemiluminescence reagent (Thermo Fisher Scientific).

Proteins interacting with SARS-CoV-2 N protein in Co-IP experiments were also analyzed by western blot analysis with anti-SARS-CoV-2 N Ab, anti-G3BP1 antibody, and anti-G3BP2 antibody, as described above.

### Confocal microscopy

Vero and Calu-3 cells were grown on glass coverslips in 12-well plates for 18 h. Cells were washed in PBS and infected with SARS-CoV-2 (0.1 MOI) for 1 h, with shaking every 15 min, in a 5% CO_2_ incubator at 37°C. The supernatants were then aspirated, and cells were cultured for 47 h or 71 h in DMEM, containing 2% FBS, followed by treatment with 0.5 mM sodium arsenite (Merck KGaA, Darmstadt, Germany), an inducer of stress granules, for 1 h. Treated and control cells were fixed with 4% paraformaldehyde in PBS, permeabilized with 0.1% Triton X-100, and blocked with 3% bovine serum albumin (BSA). These cells were incubated with anti-SARS-CoV-2 N mAb and anti-G3BP1 antibody (Cell Signaling) for 2 h at room temperature. Cells were then washed with PBS, containing 0.1% Triton X-100 and 1% BSA, and stained with Alexa Fluor 546-conjugated goat anti-mouse IgG (h + L) antibody and Alexa Fluor 488-conjugated goat anti-rabbit IgG (h + L) antibody for 1 h. Nuclei were stained with Hoechst 33258 (Thermo Fisher Scientific), and samples were analyzed by confocal laser scanning microscopy (CLSM, LSM 710; Carl Zeiss, Jena, Germany).

### Statistical analysis

Results are shown as the mean ± standard deviation. Differences between two samples were evaluated using the Student’s *t*-test, with *P* < 0.05 as the threshold for statistical significance.

## Results

### SARS-CoV-2 N protein interacts with cellular G3BP

**FIGURE 1:**
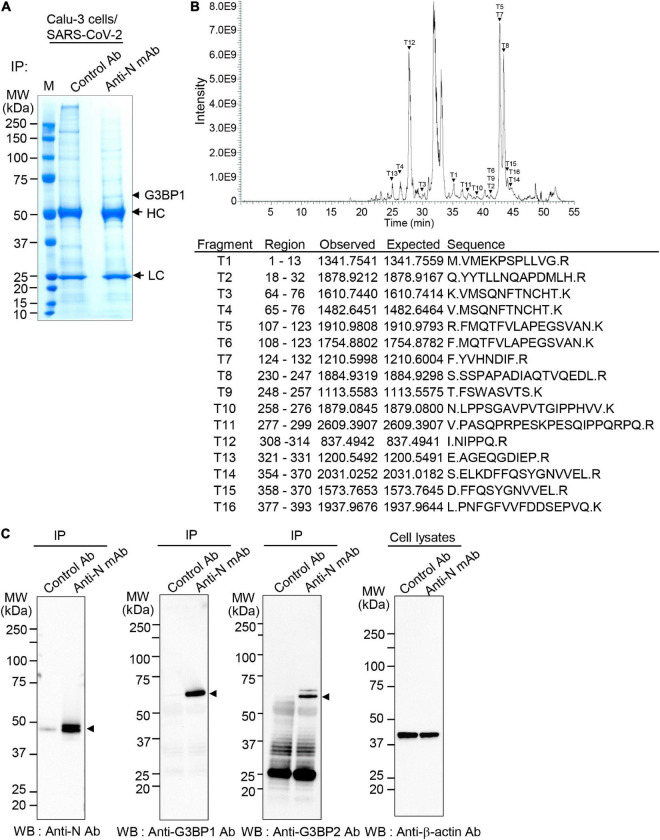
Interaction between severe acute respiratory syndrome coronavirus 2 (SARS-CoV-2) nucleocapsid (N) protein and Ras-GTPase-activating SH3-domain-binding protein 1 (G3BP1) **(A)** Co-immunoprecipitation (Co-IP) experiments to identify N protein-binding partners. Calu-3 cells were mock-infected or infected with SARS-CoV-2 S clade at 0.1 multiplicity of infection (MOI); lysates prepared at 72 h post-infection were immunoprecipitated with control antibody (normal IgG) or anti-SARS-CoV-2 N monoclonal antibody (mAb). HC, heavy chain. LC, light chain. **(B)** Identification of G3BP1. The ∼52.1 kDa protein band interacting with SARS-CoV-2 N protein in Co-IP experiments **(A)** was in-gel digested with trypsin, and the 18 peptides were analyzed by liquid chromatography-tandem mass spectrometry (LC-MS/MS). MS/MS analyses of mass peaks (arrows) reveal the peptide spectra of G3BP1. **(C)** Co-IP experiments to confirm interaction between SARS-CoV-2 N protein and G3BP1. Co-IPed immunocomplexes from panel **(A)** were subjected to western blot analysis with the indicated antibodies; anti-β-actin antibody was used as the input control. IP, immunoprecipitation; WB, western blot; Anti-N mAb, anti-SARS-CoV-2 N mAb (clone 1G10C4 mAb); Anti-N Ab, anti-SARS-CoV-2 N polyclonal Ab.

During SARS-CoV-2 infection, components of virus interact with various host cell proteins to aid in viral replication. One potential host-interacting viral component is the N protein, which acts as the dominant antigenic target for detection of SARS-CoV-2 infection (Yu et al., 2022). Here, to identify host proteins that interact with the viral N protein, we infected Calu-3 cells with the SARS-CoV-2 S clade (hCoV-19/Korea/KCDC03/2020, lineage A, NCCP43326) strain at 0.1 MOI. Calu-3 cell lysates were then immunoprecipitated with control or anti-SARS-CoV-2 N monoclonal antibody, and the associated proteins were resolved by SDS-PAGE. Results reveal interaction between N protein and a host protein with a molecular weight of ∼52 kDa (Figure 1A). This protein band was excised from the gel and analyzed by liquid chromatography (LC)-MS/MS, which identified 16 peptide fragments exhibiting amino acid sequence coverage across 41% of the full-length G3BP1 protein (Figure 1B). Thus, these data strongly suggest an interaction between SARS-CoV-2 N protein and cellular G3BP.

To further confirm binding between the SARS-CoV-2 N and G3BP proteins, Calu-3 cell lysates were immunoprecipitated with control or anti-SARS-CoV-2 N monoclonal antibody and analyzed by western blot with anti-SARS-CoV-2 N polyclonal antibody, anti-G3BP1, and anti-G3BP2 antibodies. As shown in Figure 1C, results from these Co-IP experiments show that both G3BP1 and G3BP2 are immunoprecipitated by SARS-CoV-2 N, further confirming an interaction between these proteins.

**FIGURE 2:**
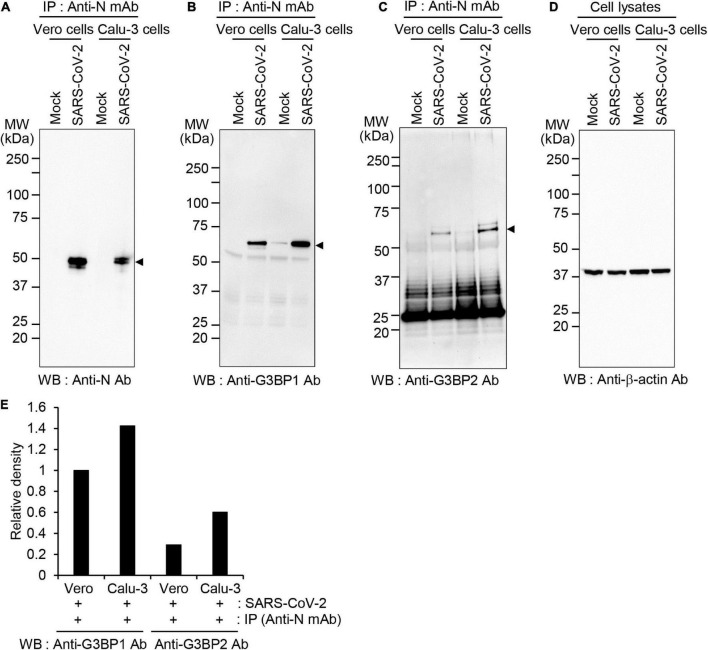
Differential association between SARS-CoV-2 N protein and G3BP1/2 in Vero and Calu-3 cells **(A–D)** Vero and Calu-3 cells were mock-infected or infected with SARS-CoV-2 S clade at 0.1 MOI; cell lysates were immunoprecipitated with anti-SARS-CoV-2 N mAb and analyzed by western blot with anti-SARS-CoV-2 N Ab **(A)**, anti-G3BP1 antibody **(B)**, anti-G3BP2 antibody **(C)**, or anti-β-actin antibody, as the input control **(D)**. **(E)** The band intensities of G3BP1 and G3BP2 were normalized with those of β-actin and the relative band intensities of Co-IPed proteins are shown on the graph. Anti-N mAb, anti-SARS-CoV-2 N mAb (clone 1G10C4 mAb); Anti-N Ab, anti-SARS-CoV-2 N polyclonal Ab.

To determine whether this interaction also occurs in other cells susceptible to SARS-CoV-2 infection, we assessed binding between SARS-CoV-2 N protein and G3BP in Vero cells and compared the binding efficiency with that observed in Calu-3 cells (Figure 2). Co-IP experiments followed by western blot analysis revealed that SARS-CoV-2 N protein is expressed to higher levels in Vero cells compared to Calu-3 cells (Figure 2A). However, a higher degree of binding between SARS-CoV-2 N protein and both G3BP1 and G3BP2 is observed in Calu-3 cells *vs*. Vero cells, as evidenced by the increased band intensity for Co-IPed proteins in this cell line (Figures 2B,C,E). Collectively, our data therefore reveal that SARS-CoV-2 N protein can bind with both G3BP1 and G3BP2, and this interaction occurs to a greater extent in Calu-3 cells than in Vero cells.

### SARS-CoV-2 infection does not affect expression of G3BP1 or G3BP2 in SARS-CoV-2-infected cells

**FIGURE 3:**
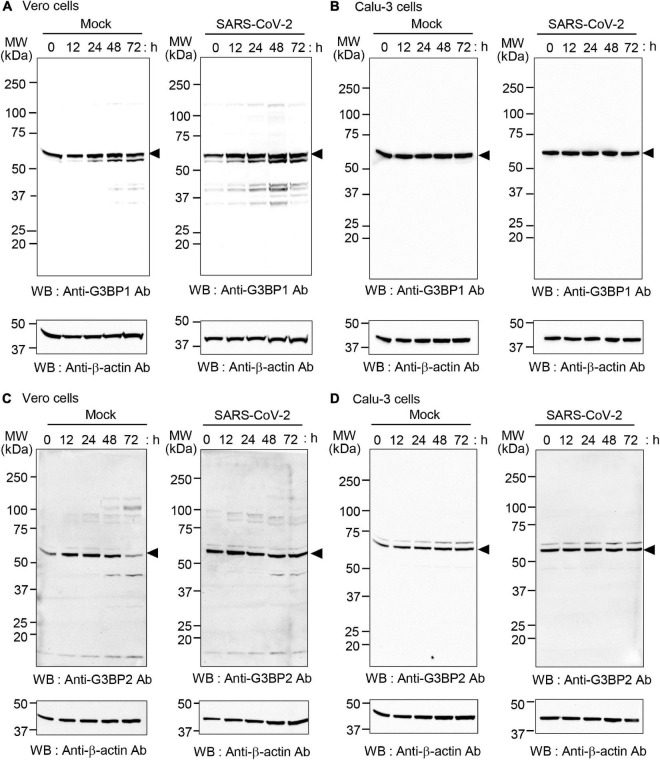
Expression of G3BP1 and G3BP2 in SARS-CoV-2-infected Vero and Calu-3 cells. Vero cells **(A,C)** and Calu-3 cells **(B,D)** were mock-infected (left) or infected with SARS-CoV-2 S clade at 0.1 MOI (right) for the indicated times. Cell lysates were prepared and analyzed by western blot with anti-G3BP1 antibody **(A,B)** or anti-G3BP2 antibody **(C,D)**. In all cases, anti-β-actin was used as the input control (shown at bottom in all panels).

The increased binding of SARS-CoV-2 N protein to G3BP1 and G3BP2 observed in Calu-3 compared to Vero cells might also result from differential expression of G3BP1 and G3BP2 in these cell lines upon SARS-CoV-2 infection. To investigate this possibility, Vero and Calu-3 cells were mock-infected or infected with the SARS-CoV-2 S clade strain at 0.1 MOI, and cell lysates were analyzed by western blot (Figure 3). Our results show that protein levels of G3BP1 are unaltered during the entire course of SARS-CoV-2 infection in both cell lines. However, protein level of G3BP2 in Vero cells was significantly reduced at 72 h in the mock infection. As we cultured the cells in medium containing 2% FBS for these experiments, the condition at 72 h may affect protein expression because of factor starvation and this can result in decrease of sensitive proteins. Considering that G3BP2 but not G3BP1 is regulated by ubiquitin-proteasome system (Soncini et al., 2001; Takayama et al., 2018; Anisimov et al., 2019) we speculate that G3BP2 protein was degraded at 72 h in mock-infected Vero cells. Calu-3 cells grow much slowly than Vero cells, therefore it is natural that expression of G3BP2 proteins was not changed during the entire culture period. Importantly, expression of G3BP1/2 was not drastically induced upon virus infection in Calu-3 cells. Thus, taken together, our data indicate that the SARS-CoV-2 N protein binds both G3BP1 and G3BP2 to a higher degree in Calu-3 cells than in Vero cells.

### Effects of SARS-CoV-2 infection on stress granule formation

**FIGURE 4:**
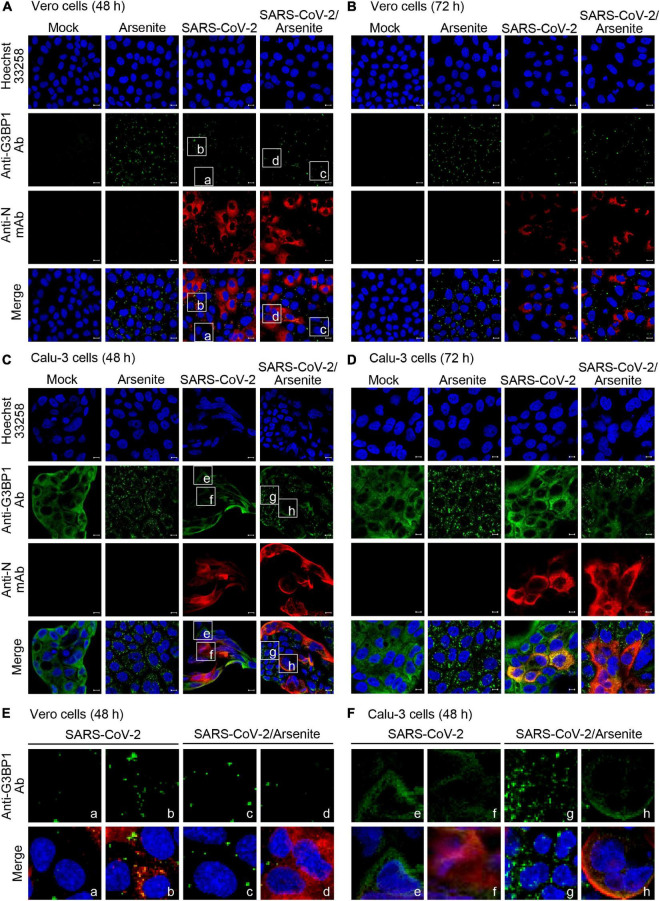
Stress granule production in SARS-CoV-2-infected cells. Vero **(A,B)** and Calu-3 cells **(C,D)** were mock-infected or infected with SARS-CoV-2 S clade at 0.1 MOI and examined by immunofluorescence confocal microscopy at 48 h **(A,C)** and 72 h **(B,D)** post-infection. Cells were treated with 0.5 mM sodium arsenite or phosphate-buffered saline (PBS) control for 1 h, prior to fixing and staining with anti-GRBP1 antibody (green) and anti-SARS-CoV-2 N mAb (red). **(E,F)** Enlarged images. The boxed regions in A and C were magnified to show the relationship between N protein expression and stress granule formation. **(E)** Vero cells: cells not expressing N protein (a, c) were compared with N protein expressing cells (b, d). **(F)** Calu-3 cells: cells not expressing N protein (e, g) were compared with N protein expressing cells (f, h). Anti-N mAb, anti-SARS-CoV-2 N mAb (clone 1G10C4 mAb); Anti-N Ab, anti-SARS-CoV-2 N polyclonal Ab. Nuclei were stained with Hoechst 33258 (blue). Anti-N Ab, anti-SARS-CoV-2 N mAb (clone 1G10C4 mAb).

Numerous viruses, including SARS-CoV-2, are known to induce formation of stress granules within infected cells. Notably, both G3BP1 and G3BP2 are required for initiation of stress granule formation, and this process can be inhibited by the binding of viral protein to G3BP1 and G3BP2 (Matsuki et al., 2013). This suggests that SARS-CoV-2 may similarly block stress granule formation via G3BP1/G3PB2 binding. To test this hypothesis and investigate the effect of SARS-CoV-2 infection on stress granule formation, Vero and Calu-3 cells were mock-infected or infected with SARS-CoV-2 S clade stain, and stress granules were visualized by immunostaining and confocal microscopy. Sodium arsenite, a common inducer of oxidative stress that promotes stress granule formation, was used as a positive control for stress granule formation (Figure 4). We found that infection with SARS-CoV-2 S clade induces the formation of G3BP1-positive stress granules in Vero cells, but not in Calu-3 cells (Figures 4A–D). Particularly, the pattern of N protein expression and stress granule formation indicate that stress granule formation occurs in SARS-CoV-2-infected Vero cells expressing the N protein (Figure 4E, left). Conversely, we detected no stress granule formation in Calu-3 cells, irrespective of N protein expression, suggesting that the robust interaction between N and G3BP proteins effectively suppresses stress granule formation (Figure 4F, left).

As expected, we further found that treatment with sodium arsenite induces significant and extensive formation of G3BP1-positive stress granules in uninfected Vero and Calu-3 cells (Figures 4A–D). Intriguingly, however, this sodium arsenite-induced stress granule formation is suppressed by SARS-CoV-2 S clade infection in both Vero and Calu-3 cells, indicating that virus infection can inhibit the formation of stress granules in response to other stressors (Figures 4A–D). Enlarged images clearly show suppression of arsenite-induced stress granule formation in N protein-positive cells (Figures 4E,F, right). These data indicate that SARS-CoV-2 can differentially regulate stress granule formation induced by viral infection and sodium arsenite in Calu-3 cells and Vero cells.

### Effects of SARS-CoV-2 variants on stress granule formation

**FIGURE 5:**
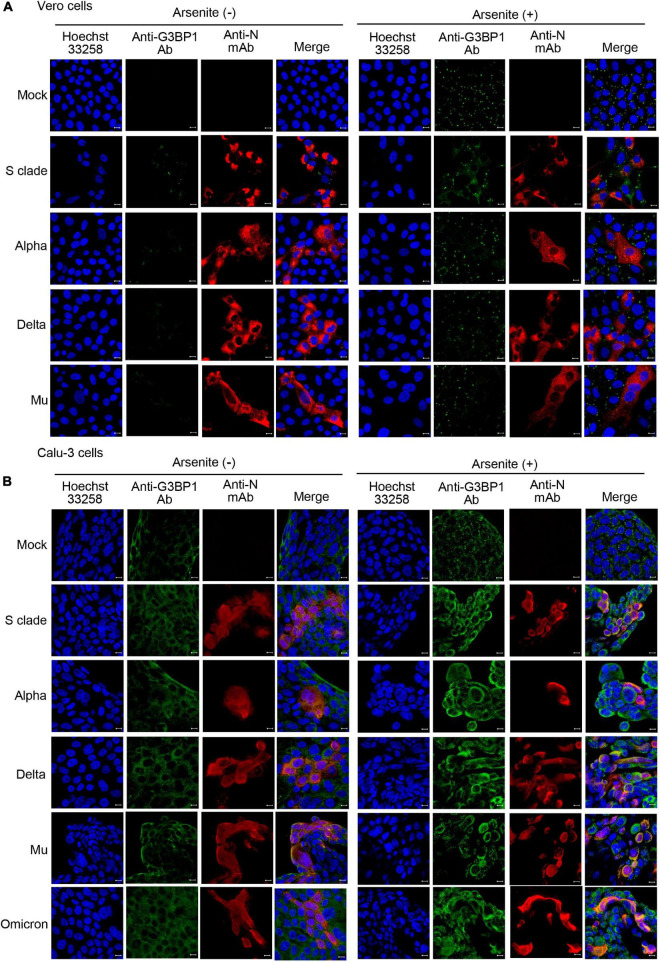
Detection of stress granules in cells infected with different SARS-CoV-2 variants. Vero cells **(A)** and Calu-3 cells **(B)** were mock-infected or infected with SARS-CoV-2 variants at 0.1 MOI for 71 h and analyzed by immunofluorescence confocal microscopy. Cells were treated with PBS or 0.5 mM sodium arsenite for 1 h, prior to fixing and staining with anti-GRBP1 antibody (green) and anti-SARS-CoV-2 N mAb (red). Nuclei were stained Hoechst 33258 (blue). Anti-N Ab, anti-SARS-CoV-2 N mAb (clone 1G10C4 mAb).

Infectivity and propagation rates of different SARS-CoV2 strains vary greatly, likely contributing to the differential severity of various waves observed during the COVID-19 pandemic. Similarly, inhibition of the stress granule formation might also vary in different SARS-CoV-2 strains and affect viral pathophysiology in a strain-specific manner. We therefore measured the effect of infection with other SARS-CoV-2 variants on stress granule formation. We found that infection with SARS-CoV-2 alpha, delta, or Mu variants induces stress granule formation in Vero cells but not in Calu-3 cells (Figures 5A,B; left panel). Moreover, SARS-CoV-2 alpha, delta, and Mu variants inhibit sodium arsenite-induced stress granule formation similar to SARS-CoV-2 S clade in both Vero cells and Calu-3 cells (Figures 5A,B; right panel). Taken together, these results suggest that stress granule formation in response to SARS-CoV-2 infection might be guided by the host cell intrinsic factors rather than those of viral proteins.

## Discussion

The ongoing COVID-19 pandemic has caused significant mortality and financial adversity worldwide, highlighting the urgent need for an improved understanding of the mechanisms that drive the SARS-CoV-2 life cycle, including those involved in infection, replication, and transmission. Numerous viruses have been shown to withstand stress granule formation, often by inhibiting their formation via the targeting of stress granule-nucleating proteins, particularly G3BP1 (Kang et al., 2021). SARS-CoV-2 appears to be one such virus, with recent studies reporting that the SARS-CoV-2 N protein targets both G3BP1 and G3BP2, thereby attenuating stress granule formation and promoting viral replication (Zheng et al., 2021; Nabeel-Shah et al., 2022). However, it is not known whether there are differential effects of SARS-CoV-2 infection on stress granule regulation in different cell types.

In this study, we addressed this question by using our previously developed anti-SARS-CoV-2 N antibody to measure the binding of SARS-CoV-2 N protein to cellular G3BP1 in two different cell lines (*i.e.*, Vero and Calu-3 cells). Notably, we found that SARS-CoV-2 N protein interacts with both G3BP1 and G3BP2 proteins in Calu-3 and Vero cells, although this binding is present to a greater extent in Calu-3 cells relative to Vero cells. Further analysis revealed that levels of G3BP1 and G3BP2 do not change during SARS-CoV-2 infection in both cell lines, confirming that the observed differences are due to more robust binding between SARS-CoV-2 N and G3BP1/2 in Calu-3 cells and not differential expression of these proteins in different cells types. Consistent with these observations, Calu-3 cells infected with SARS-CoV-2 do not display stress granule formation, whereas Vero cells show a moderate degree of stress granules upon SARS-CoV-2 infection. Therefore, our data suggest that N protein binding to G3BP1 and G3BP2 with N protein negatively regulates stress granule formation.

SARS-CoV-2 variants display various degrees of transmissibility and confer differing levels of disease severity in infected patients (Harvey et al., 2021; Mendiola-Pastrana et al., 2022). Moreover, studies have shown that SARS-CoV-2 variants have different replication rates and produce distinct viral titers from infected Vero cells (Jeong et al., 2021; Prince et al., 2022). Because inhibition of stress granule formation is related to viral replication (McCormick and Khaperskyy, 2017), we hypothesized that SARS-CoV-2 variants might also show differing degrees of stress granule inhibition. We therefore measured stress granule formation in Vero and Calu-3 cells infected with SARS-CoV-2 variants and further determined how these affect sodium arsenite-induced stress granule formation. Our results revealed similar patterns of stress granule formation and regulation in cells infected with the different viral strains, suggesting that extent of stress granule inhibition is not always reflective of virus production efficiency. In addition, although stress granule formation in response to SARS-CoV-2 infection is more prominent in Vero cells, with weaker binding between N protein and G3BP1/2, viral production is also higher in these cells relative to Calu-3 cells. Contrary to these findings, however, a previous study reported that knockdown of G3BP1 inhibits stress granule formation and enhances replication of the SARS-CoV-2 genome in Hela-ACE2 cells (Zheng et al., 2021). Collectively, these data suggest that SARS-CoV-2 production is only partly regulated by stress granule formation, and this phenomenon is largely determined by host cell intrinsic properties. However, a detailed mechanistic study using an array of different cell lines and viral variants will be required to fully elucidate the complex relationship between stress granule formation and SARS-CoV-2 virus replication.

In summary, utilizing two susceptible cell lines and various natural SARS-CoV-2 variants, we found that inhibition of virus-induced stress granule formation correlates with binding between N protein and G3BP1/2; however, it does not always reflect the degree of virus production or ability to inhibit stress granules induced by sodium arsenite treatment. These findings highlight a clear gap in knowledge relating to the role of stress granules in SARS-CoV-2 infection and suggest that more studies are needed to determine whether they may represent a viable target for COVID-19 treatment.

## Data availability statement

The datasets presented in this study can be found in online repositories. The names of the repository/repositories and accession number(s) can be found below: ProteomeXchange, PXD035715.

## Author contributions

H-JK and YL conceived of the project. H-JK, JGS, and YL designed the experiments. H-JK, SM, and YL wrote the manuscript. DK, SM, MKa, JK, SP, MKi, KB, and SK carried out the experiments. H-JK, DK, JGS, and YL analyzed the data. All authors approved the final version of the manuscript.

## Footnotes

1
http://www.ncbi.nlm.nih.gov
